# Change of Location

**Published:** 1916-05

**Authors:** 


### Change of Location.

Dr. S. S. Robinson, of Dallas, has moved to Rosebud, where he will practice his profession.

Dr. J. J. Jeter and family, of Phalba, have moved to Wills Point.

Dr. L. V. Dawson, of Odessa, Mo., has moved to Plainview, where he will practice medicine and surgery.

Dr. N. D. Carter, of Harris, Ark., has moved to Robstown, Texas.

Dr. L. A. Patterson and wife have moved to Utopia from Medina.

Dr. J. J. Jeter, of Phalba, who has been practicing medicine in the southern part of Van Zandt and in Henderson county for the past four years, has moved to Wills Point, where he will be associated with his brother, E. D. Jeter, in the practice of medicine.

Dr. A. J. Sharp and family, of Franklin, have moved to Pottsboro.

Dr. and Mrs. John Harrison, of Illinois, have located permanently in La Porte.

Dr. and Mrs. Copeland, physicians from Loraine, will make their future home in Pecos, where they will practice their profession.

Dr. H. S. Cearnal has moved to Sherman from Missouri. He goes to Sherman highly recommended as a physician and gentleman.

